# Teaching Students About Plagiarism Using a Serious Game (Plagi-Warfare): Design and Evaluation Study

**DOI:** 10.2196/33459

**Published:** 2022-02-16

**Authors:** Abejide Ade-Ibijola, Keagan Young, Nashik Sivparsad, Mpho Seforo, Suhail Ally, Adebola Olowolafe, Maria Frahm-Arp

**Affiliations:** 1 Johannesburg Business School University of Johannesburg Johannesburg South Africa; 2 Library and Information Centre University of Johannesburg Johannesburg South Africa

**Keywords:** serious games, educational games, plagiarism, library games, game mechanics, education, teaching

## Abstract

**Background:**

Educational games have been proven to support the teaching of various concepts across disciplines. Plagiarism is a major problem among undergraduate and postgraduate students at universities.

**Objective:**

In this paper, we propose a game called Plagi-Warfare that attempts to teach students about plagiarism.

**Methods:**

To do this at a level that is beyond quizzes, we proposed a game storyline and mechanics that allow the player (or student) to play as a mafia member or a detective. This either demonstrated their knowledge by plagiarizing within the game as a mafia member or catching plagiarists within the game as a detective. The game plays out in a 3D environment representing the major libraries of the University of Johannesburg, South Africa. In total, 30 students were selected to evaluate the game.

**Results:**

Evaluation of the game mechanics and storyline showed that the student gamers enjoyed the game and learned about plagiarism.

**Conclusions:**

In this paper, we presented a new educational game that teaches students about plagiarism by using a new crime story and an immersive 3D gaming environment representing the libraries of the University of Johannesburg.

## Introduction

Plagiarism is the reuse of another person’s ideas, work, or words as one’s own, without sufficient credit being given to the original creator [1]. Reasons people plagiarize are that the benefits outweigh the drawbacks, while a student’s motivation to plagiarize could be the goal of good grades and the ability to measure up with their peers [2]. Another noteworthy reason is that with the flood of online information, several researchers have noted that students are more likely to ignore the ethics behind plagiarizing [3].

Beyond concerns in the student realm is a slight, less rampant concern with academics who also plagiarize in their own academic writings [4]. This is because certain research institutions offer financial incentives/subsidies (sponsored by the government of the country) for research outputs, making academics in such institutions more desperate to produce more research [4]. Although most academics do not plagiarize, they sometimes look the other way when their students do. One, because of the concerned modules [5], and sometimes because of the stressful red tape that goes into reporting these transgressions to the institution, as this leads to many meetings and validation processes [6,7]. If students are trained well, and taught what is and is not plagiarism, they often avoid it [8,9]. Many of the incidences reported are attributed to ignorance on the part of the student, the incapability to write original content, the pressure on postgraduate students to publish, or simply the lack of realization and understanding of what is implied by plagiarism [8]. In this paper, we propose a game called Plagi-Warfare (a video exhibition of Plagi-Warfare has been created earlier [10]) that attempts to teach students about plagiarism.

A number of software tools or technological solutions have been proposed to assist in the fight against plagiarism. Popular paid tools (such as those listed in Tripathi et al [11]) include Turnitin, CopyCatch Gold, and EVE2: Essay Verification Engine, while some free online tools include Dupli Checker, Copyleaks, and PaperRater. Particularly, games have also been created to engage students or players with plagiarism educational contents. Here, we briefly mention 3 of these games. The first is Goblin Threat [12]. Goblin Threat is played by finding and clicking on goblins, who ask questions about different aspects of plagiarism. Sound and engaging design are used to keep the players’ attention. The second is Cheats and Geeks [13], a board game where players race against a rival to display their research. Players are enticed by a chance to move faster by committing wrongdoings. Finally, Planet in Peril [14] is a game where the player navigates through a 3D campus environment and learns about plagiarism through exchange with aliens. There are several opportunities to improve existing educational games for teaching plagiarism or other subjects/contents. First, it is helpful to look at the limitations of existing games and attempt to address those limitations in creating more effective games for teaching a topic such as plagiarism. Some of such limitations are as follows:

- Some of these games are especially relating to students in certain academic fields [14]. This makes the audience restricted. A game that will teach students about plagiarism should be playable by students who are not gamers.
- According to Broussard [14], many educational games are not engaging enough to allow players to select their own in-game visual representations (or avatars), stating that this could improve the player’s compassion for their virtual self.
- Kazimoglu et al [15] raised a similar concern about games not having multiple gameplay paths, forcing the player to make similar choices every time, hence leading to boredom for the player.
- As far as we know, no library plagiarism game takes place in a virtual environment that its students are familiar with, within the context of South African university libraries.
- The levels of complexity of gameplay are not well defined (and mostly absent) in these educational or plagiarism-teaching games [16].
- Due to the static nature of the contents of plagiarism and the challenge of presenting this content using the vehicle of games, previous games lack the replayability factor—gamers quickly get used to what challenges pop up in the game and where—leading to boredom after a few iterations of playing the game [17-19].
- The storyline of some of the previous games is mostly unrelatable to the students, for example, having aliens on campus in the game Planet in Peril [14]. We have evidence to show that games having realistic stories are more relatable to students [20].

This paper is organized as follows. The Methods section presents the game design and algorithms for the new game that teaches plagiarism, Plagi-Warfare, as well as the technologies used in the implementation of Plagi-Warfare. Discussed in the Results section is an evaluation on the feedback on Plagi-Warfare. The Discussion section presents the background of the work and an analysis of related works, with the gap that is addressed in this work. Finally, we present the conclusions of this work and future works.

## Methods

**Figure 1:**
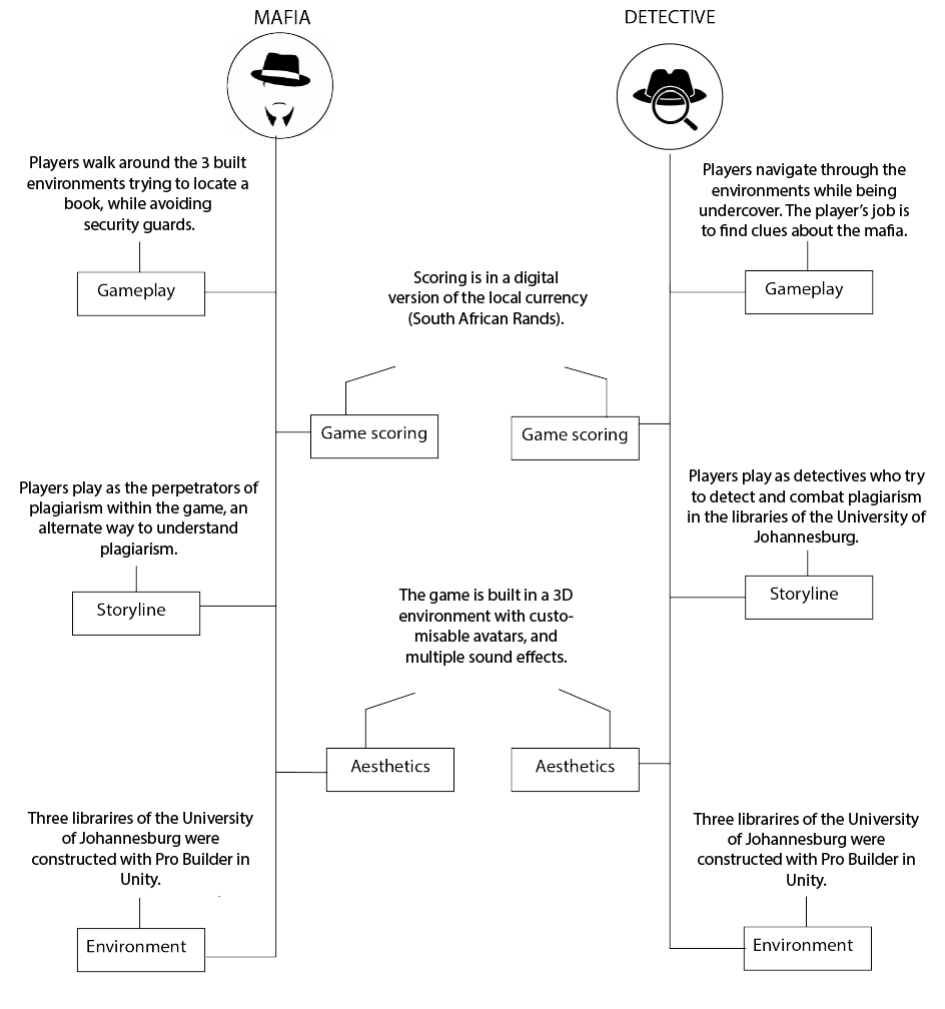
Plagi-Warfare game components.

In this section, we present aspects of the game design of Plagi-Warfare. We start with the writing of the game’s story, followed by the description of the world where the story takes place. We end it with the description of the content of the game (plagiarism lessons, quizzes, or facts), levels of the game, scoring in the game, the technological/software design of the system for the game, and the follow of the game. Figure 1 shows the Plagi-Warfare game components.

### Story Development

Here, we present the background story of Plagi-Warfare. In Plagi-Warfare, the player is able to choose whether to play as the bad guy or the good guy, that is, as a *mafia member* or a *detective*. The story of the mafia is that the player is an expelled student who is back to assist students in plagiarizing. The player joins the *plagiarism cartel* as a low-ranking member, completing tasks sent to their virtual phone and avoiding getting caught by detectives. The detective mode allows the player to be hired to investigate plagiarism cases within the library. The player walks around the library, questioning students about their work and assignments, to find more out about the plagiarism mafia. The students found guilty are questioned further and informed about the consequences of their actions. The player then uses what they have found to take down the plagiarism mafia.

### World Design

In this section, we describe the design of the game world or environment. The environments in Plagi-Warfare were built using Unity Game Engine; it plays out in 3D gaming environments that are modeled after the libraries of the University of Johannesburg, South Africa. The libraries used in the gaming environment include the Auckland Park Bunting Road (APB) campus, the Kingsway Avenue (Auckland Park Kingsway [APK]) campus, and the Doornfontein (DFC) campus libraries. In Plagi-Warfare, there are 27 scenes: 4 of the scenes are used to assist the player to sign into/up for the game; 5 of the scenes are for the navigation for each mode of gameplay (resulting in 10 scenes for the mafia and detective modes)—that is, used to create pages for the user to access the leaderboard, options, profile, level selection, and level failed message; 7 scenes are for quizzes or game challenges; and 6 scenes are library environments, 3 for each game mode, representing the 3 libraries of the university.

### Content Design

The content of Plagi-Warfare are the questions or challenges in the game. The questions that constitute challenges in the game were supplied by the librarians at the University of Johannesburg. These challenges are meant to test the players’ knowledge about plagiarism through scenario-based questions. The questions are composed with Boolean responses (true or false), identifying the best answer from multiple answers and identifying odd answers, word responses, and short text responses. To make sure that Plagi-Warfare is replayable, we used the seed questions/challenges to create an algorithm for the procedural generation of similar instances of the same questions, allowing the questions to be presented in a slightly different way every time. This has been proven to keep the interest of players in the game [21,22].

### Level Design

There are 6 levels in Plagi-Warfare. In the mafia role and in the detective role, there are 3 levels. The mafia levels are divided into 2 types of activities: The first type of activity involves the players exploring the gaming environment (which are virtual libraries) with the controllers and engaging in maneuverings, such as avoiding patrolling detectives (because the detectives are on the lookout for mafia members), while trying to locate hidden objects, such as books. The second type of activity is where the knowledge of the player about plagiarism is tested using scenarios or quizzes. The detective levels are divided into 2 types of activities: The first type of activity requires the player to roam the virtual environment and speak to student nonplayer characters (NPCs) to find potential mafia members. The second type of activity is to interrogate suspicious student NPCs with the use of a quiz. Players have to determine whether the student NPCs’ answers are correct or incorrect. The hardware required to play Plagi-Warfare is a desktop or a personal computer with an attached mouse and keyboard.

The following mechanics are used to control the level of difficulty in Plagi-Warfare:

- Each time a level is loaded, the mission objective’s location is randomized. This forces the player to search through the whole environment, still having the risk of being caught by detective NPCs.
- The detectives’ alert proximity increases as the levels progress, which increases the chances of the player getting caught.
- There is an increase in the difficulty or complexity of questions used in the quizzes.
- The number of questions asked increases in the game as the level increases.

The average range of completion time for each level of Plagi-Warfare is 5-10 minutes.

### Scoring Design

Plagi-Warfare is scored in South African Rands (symbolically noted as ZAR or simply R). Players are allocated virtual money for completing levels of the game. For each question answered correctly, a player gets R250 ($16.38). If a player answers 6 correct questions in a level, they are given R1500 ($98.27) for that level. Players are expected to gain bragging rights from their net worth in the game. For a player to proceed, they have to answer 80% of a level’s questions. There is a leaderboard in the game that shows the top players and their respective net worth.

### Game Flow Design

**Figure 2:**
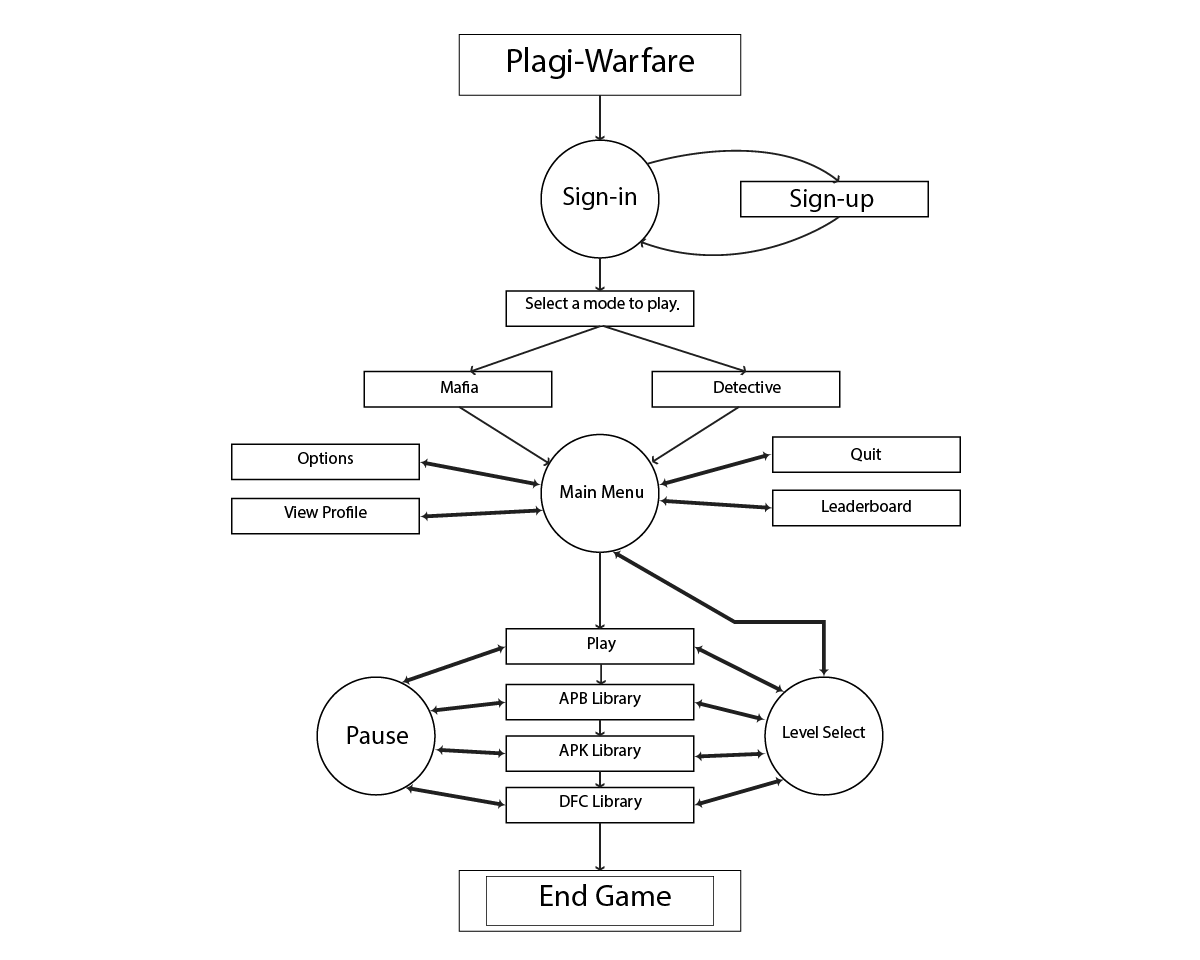
Plagi-Warfare game flow design. APB: Auckland Park Bunting Road; APK: Auckland Park Kingsway; DFC: Doornfontein.

The flow of play in Plagi-Warfare is illustrated in Figure 2. The player starts by signing in (or signs up if the player is new to the game), and they select the desired mode of play, detective or mafia member. The player is then presented with menu options to access the leaderboard and game options (where they can select avatars to represent themselves), view their profile, or select a level to play. When all levels are completed for the selected mode, the game halts—“End Game.”

### Design of Players’ Feedback

We selected 30 participants from the University of Johannesburg across different backgrounds, all having good exposure to games and a good knowledge of what plagiarism is. The following are the questions from the survey:

- How likely would you play the game? (1, unlikely; 10, extremely likely)
- Would you recommend the game to someone else to play?
- Do you think this game can help in educating students about plagiarism?
- What would you enjoy about this game?
- What would you improve about the game?

### Implementation, Results, and Evaluation

Here, we present the technologies used for the implementation of Plagi-Warfare, a description of some important algorithms designed to perform certain tasks (such as procedural content generation of variants of plagiarism quizzes), results in the form of screenshots from the finished game, and an evaluation showing the opinion of players of this game.

### Systems Design, Tools, and Resources

**Figure 3:**
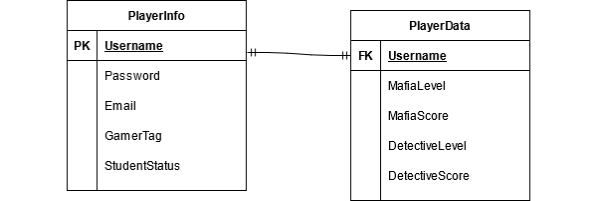
Plagi-Warfare database design.

Plagi-Warfare was created using the Unity Game Engine, alongside the Microsoft Visual Studio Integrated Development Environment (IDE). We also used some asset libraries, such as Unity Asset Store, Turbosquid, and Cgtrader (used for both 3D models and 3D graphics) and Mixamo (used for character animation and generation). The database is hosted on PhpMyAdmin, which holds 2 tables, PlayerInfo and PlayerData. PlayerInfo holds the account information of the player—that is, username, email, password, gamer tag, and student status. PlayerData holds the game data of the player—that is, current level and scores for each respective game mode. PlayerInfo has a mandatory one-to-one relationship with PlayerData. A simple entity-relationship (E-R) model that describes these tables and their relationship is shown in Figure 3. The E-R model is a tool used by analysts to visualize data stored in a database [23].

### Algorithms

Here, we present a few algorithms that were designed to perform specific operations in the game. Algorithm 1.1 (Textbox 1) takes a set of 20 predefined locations from design time and a book object that needs to be placed in the game scene. The algorithm computes a random location for the placement of the book and returns this location to other parts of the game.

Other algorithms include the following:

- Content generation algorithm: An algorithm that takes a library quiz designed by a librarian and automatically generates variations of the quiz, thereby maximizing the replayability of the game.
- Field-of-view (FoV) algorithm: During the mafia gameplay, there are detectives patrolling the environments to attempt to catch the player. This algorithm sets an invisible radius around each detective with a set degree range and length. Should the player enter this area, a caught screen is triggered, causing the player to have to restart the level.
- Transferring NPCs: In the detective side, when the player is in range of the NPC’s box collider, this algorithm is triggered. The player is given an option on whether they want to take the NPC to the interrogation room by pressing the E key. Once this event is triggered, 3 different locations are checked. If the first location is not filled, then the NPC is moved to that position. Once all 3 locations are filled, this algorithm does not run. When the player is in close proximity to a student NPC, they are given an option to take the student NPC to the interrogation room.
- Selective shuffling: This algorithm selects random quizzes from the repository of generated quizzes, given that the player has not previously been presented with the actual instance of the quiz problem.
- Updating levels in the database: This algorithm saves the player’s current state (level of play, current score, etc) in the database.
- Answer submission: This algorithm validates the player’s answer with respect to the predefined model answer of the plagiarism quiz. Scores are awarded as a result.

**Algorithm 1.1.**

**Algorithm 1.1:** Shuffling spawn locations of objects

**Data:** LocationsXYZ [20], BookObject

**Result:** Book Location

     begin

     instance_location ← LocationsXYZ(getRandom[1,20]);

     Book_Loc ← Transform (BookObject, instance_location);

     **return** Book_Loc

end

## Results

### Screenshots From Plagi-Warfare

**Figure 4:**
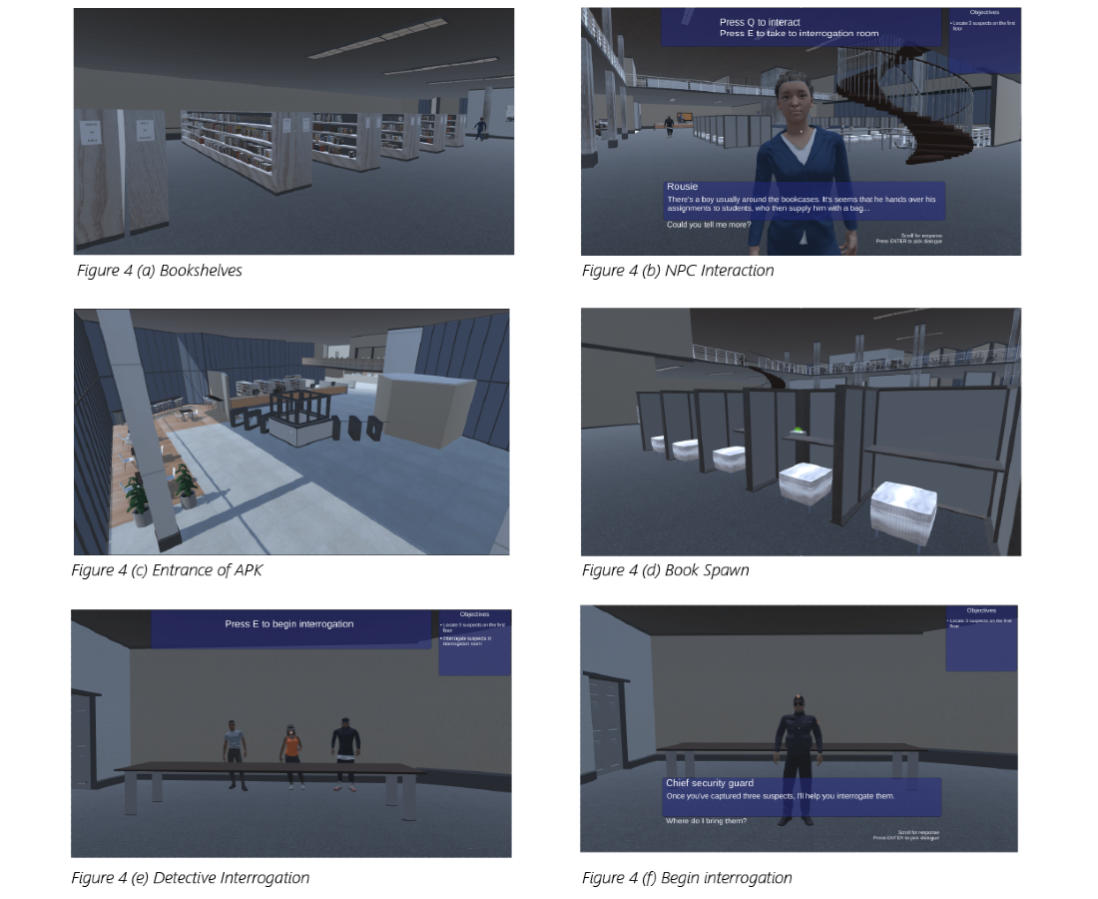
Screenshots of both gameplay and behind the scenes of environments. APK: Auckland Park Kingsway; NPC: nonplayer character.

**Figure 5:**
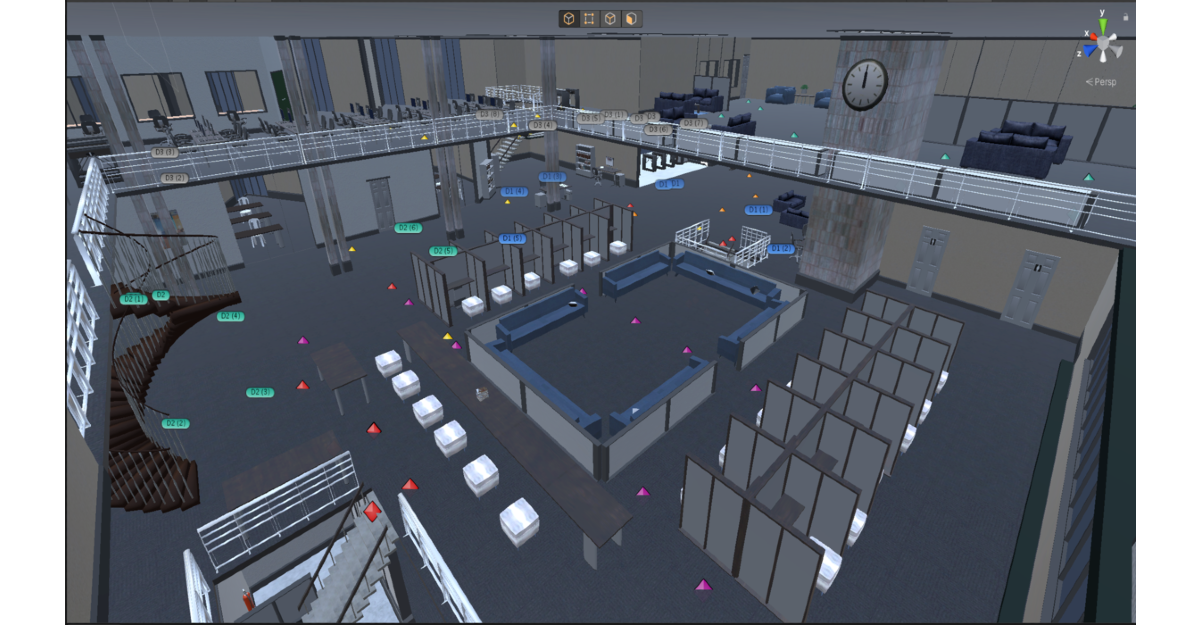
Library at APB campus, illustrating the different NPC waypoints. APB: Auckland Park Bunting Road; NPC: nonplayer character.

Here, we showcase a few screenshots from Plagi-Warfare. In Figure 4(a), the bookshelves at the APB campus library are shown. Figure 4(b) shows a conversation scene between the player and the NPC. The entrance of 1 of the environments is shown in Figure 4(c). Figure 4(d) shows 1 of the book spawn locations during a game session. The NPC interrogation is shown in Figure 4(e). In Figure 4(f), we show the detective’s introduction scene. Figure 5 presents a view of the APB library from upstairs and the different way points of the NPCs. Each color represents a different NPC path. More results (a recorded video of the game during play) are published online.

### Game Evaluation

**Figure 6:**
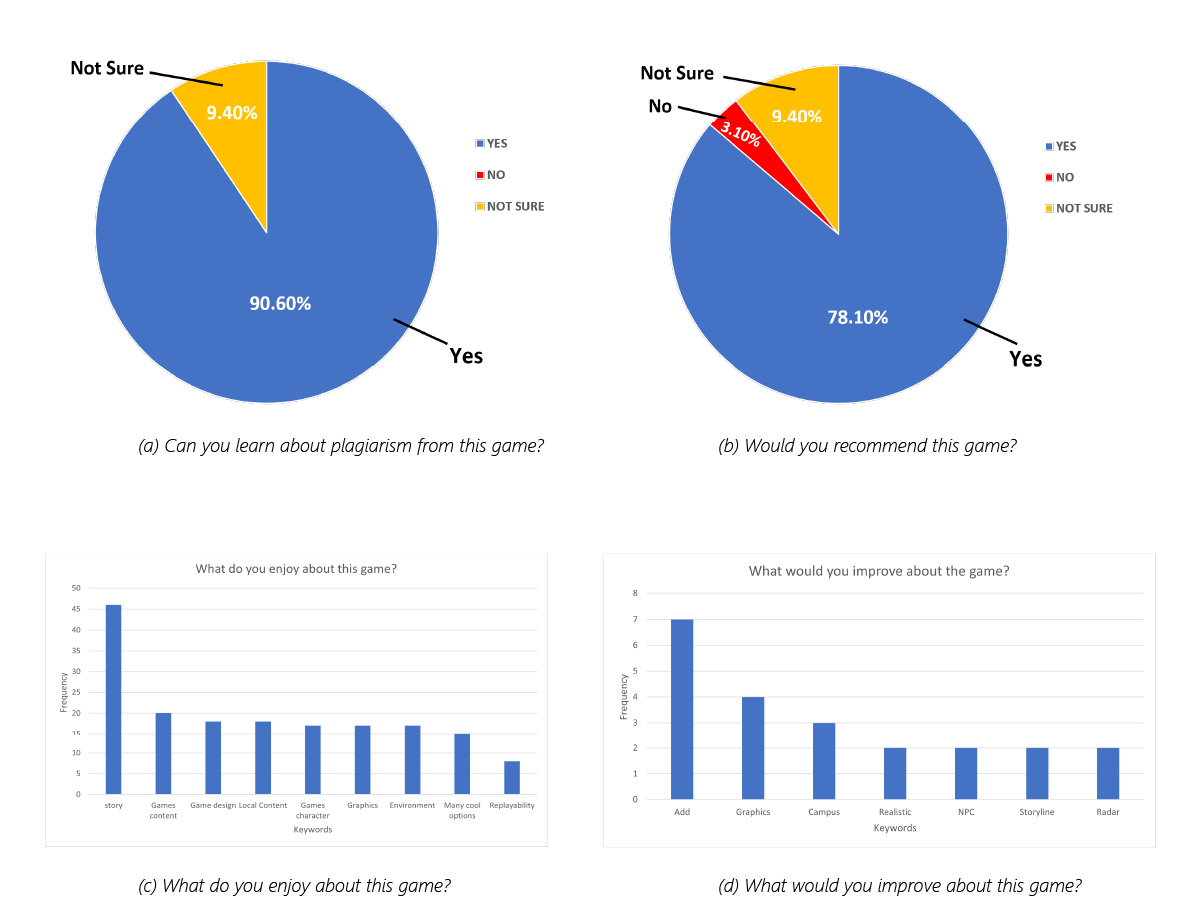
Evaluation of Plagi-Warfare. NPC: nonplayer character.

In this section, we present the result of an evaluation conducted to gather the opinions of players. The results from the evaluation are shown in Figure 6(a) and Figure 6(b). The results show that the majority (27/30, 90%) of the players found the game educational and believed that they could learn about plagiarism from the game, while a small number stated that they were not sure—there was no negative stance on this question. In addition, 1 (3%) player said that they would not recommend the game, while 23 (77%) said they would, and the remaining (6, 20%) were not sure. This is a high recommendation rate.

We also allowed the players to respond to 2 questions in free text: what they enjoyed about the game and how they think Plagi-Warfare can be improved. As seen in Figure 6(c), notable keywords that our participants repeatedly used are “story,” “game content,” and game design”; after inspecting the actual text of responses, we confirmed that this directly translates to the fact that our players loved the story, the learning that takes place, and the look and feel of the game. Other keywords were “local content” (implying that they know the library and they enjoyed that). There was little attention given to the replayability of the game—this is understandable as it only becomes important after players play for a longer time. The last question that players had to answer was whether they had other ways of improving the game. Here, many keywords came up in the responses, implying that there is no one aspect of the game that is majorly deficient. However, notable suggestions (when we eliminate obvious keywords) included “realistic” and “add/adding” (suggesting the addition of more features). These responses are good for a first launch of Plagi-Warfare and encourages a number of possible upgrades.

## Discussion

### Principal Findings

The role of serious games in the learning domain is rapidly growing. This paper explored the development of effective serious games with regard to plagiarism. We hope readers will obtain a general understanding of how to create an effective serious game to combat plagiarism.

The players found the gameplay and storyline quite engaging. This is important as serious games need to find a balance between entertainment and education in order to create an effective learning environment [24].

We explained how to develop a game that is both enjoyable and educational by demonstrating functional mechanics.

This is critical since players are less likely to learn from it if they dislike playing the game [19]. Serious games must therefore be enjoyable in order for learning to take place.

### Serious Games in the Learning Domain

In this paper, we leveraged the usefulness of serious games in the learning domain—that is, educational games—and explored new stories, gameplay, and game mechanics to present plagiarism as content in an immersive library gaming 3D environment. This resulted in a video game that we named Plagi-Warfare. In Plagi-Warfare, players can assume 2 different roles: as a mafia member or as a detective. As a mafia member, they make cash from plagiarizing for other students, and as a detective, they try to catch an NPC who is playing as a mafia member. Hence, the major contributions of this paper are as follows:

- We proposed a new game story and designed new game mechanics for student players to get into an immersive gaming environment and get rewarded by either detecting plagiarism scenarios (as detectives) or play the role of a mafia member to commit plagiarism within the game—another way of clearly acknowledging what plagiarism is.
- We created a game that gets played out in a 3D environment, representing the university library of our home university—the University of Johannesburg.
- We used new algorithms to create procedurally generated questions that are variations of typical plagiarism-related questions, ensuring replayability of the game.
- We proposed several algorithms for the implementation of this game and named this game Plagi-Warfare.
- We evaluated this game by asking students to play the game and assess its effectiveness.

### What Is Plagiarism?

The main concept of plagiarism is presenting or using someone else’s work or ideas as your own work, not citing or giving credit to the person whose idea you are using [25]. There are 2 types of plagiarism, intentional and unintentional [26]. Intentional plagiarism is when the person knows the concept of plagiarism and has the writing and academic knowledge to prevent/avoid plagiarism by correctly citing the sources but still makes the conscious decision to copy [27]. Unintentional plagiarism is more common; this is when the person who is copying has little to no understanding of the concept, has a lack of writing skills regarding citing, and a lack of knowledge about avoiding plagiarism [28].

### Plagiarism Due to Students’ Lack of Information

Unintentional plagiarism can be caused by a lack of knowledge about plagiarism [29-31]. According to Baker-Gardner et al [28], a possible determinant of whether students will plagiarize can be the students’ awareness of the plagiarism policy of the university. When plagiarism policies are clear and precise, there will be caution in trying to plagiarize as students will be aware that consequences exist [32]. Once plagiarism policies are known to not be compromised, the students will be more careful when writing in order to make sure that they do not plagiarize [28]. Another problem associated with ignorance and plagiarism is the thin line between general knowledge and authorship [33]. Being able to differentiate between common knowledge and someone’s ideas is important for students in order to avoid plagiarizing [33,34].

Although universities often provide information about plagiarism on their websites and from their libraries, we still have cases of plagiarism among students [35]. There may be documentation on plagiarism; however, students are faced with the challenge of having to read so much, so it is often difficult to identify what is significant, let alone comprehend and internalize that critical information [36]. According to the study conducted by Obeid et al [37], when students were exposed to a plagiarism intervention session (students who were exposed to plagiarism lessons), they were proven to plagiarize less in comparison to those who were in the control group (students who did not attend the plagiarism lessons). This signaled that the intervention was an efficient method of reducing plagiarism among research course students.

### Serious Games in Teaching: Educational Games

Serious games are significantly important in education and in improving the willingness of students to learn [38,39]. More satisfaction and enjoyment of the game lead to an enhanced level of interest in the subject matter for the player [40,41]. It has been shown that:

- Having a well-designed educational game and learning methods that are progressive has been a huge instrument in educational support [42].
- A test to see whether a game would improve the knowledge of the player was proven to be effective [43-45].
- Knowledge can be improved through observation and experimentation in the gaming environment [46-48].

One challenge with educational games is that the immersive part of these games is mostly missing [49,50], making these games boring. It is therefore important to find a balance between the difficulty of the game and the enjoyment of the player. Different individuals learn in different ways; hence, it is best to cater for as many learning styles as possible [51]. We know from the literature that when you combine learning activities with immersive media, it is proven to have great outcomes, sometimes even better than those in both nonimmersive and in-person circumstances [52]. Educational games have to solve this conundrum of being educational and fun/immersive in order to successfully induce learning [53,54]. An attempt to introduce balanced fun and learning was presented by Ade-Ibijola and Aruleba [24]. This motivates the creation of immersive educational games, such as the one presented in this paper—Plagi-Warfare.

### Problem Statement and Motivation

Higher education institutions around the world have a significant problem with plagiarism [55,56]. Resources, such as the internet, have contributed to an increase in plagiarism [57]. This problem became more prevalent during the 2020 COVID-19 pandemic, where all assessments were conducted online [58], leading to the emancipation of many proctoring systems [59]. There is also a concern that in most cases, students are not informed of universities’ policy on plagiarism [60]. Gullifer et al [61] stated that the amount of information students have to consume on admission to institutions is usually too much, leading to less attention on the topic of plagiarism.

Several attempts have been made to educate students about plagiarism using games [12-14]—most of these games are designed with stories, mechanics, and gameplay that the students do not find interesting or realistic [62]. This is the problem addressed in this paper—to create a more realistic story, mechanics, and gameplay in delivering an immersive educational game for teaching students about plagiarism. This we have done in a new game—Plagi-Warfare. In the following section, we discuss related works, in particular existing educational games for teaching plagiarism and other library-created games.

### Related Works and Gaps

In this section, we present related works, specifically serious games in education or educational games for teaching plagiarism and games used by libraries around the world to teach specific subjects or topics.

#### Existing Plagiarism Educational Games

Table 1 describes 4 educational games that are specifically designed to train/inform the player about plagiarism, and Table 2 lists existing library games worldwide.

**Table 1:** Existing plagiarism educational games.

Game name	Similarity to Plagi-Warfare
Cheats and Geeks [63]	Designed in the style of a dice board game, the game places the player in the role of a graduate student who has to publish a paper for a competition. The player must complete pop quizzes about plagiarism before being able to complete the challenges in the game.
Frenetic Filing [63]	A retro-style-designed game in which the player needs to solve as many processed plagiarism cases before a set time elapses. The game rewards the player with virtual sneakers and coffee that assist in speeding up the player’s review of cases.
Murky Misconduct [63]	Murky Misconduct is a crime detective game where the player engages as a plagiarism investigator. They are tasked with tracking down potential perpetrators by comparing academic papers and finally prosecuting the players. Players are educated on the complex ethics issues and consequences of plagiarism.
Goblin Threat [12]	This game is designed as a clicking game, where the player must find hidden goblins, which ask the player questions related to plagiarism. Game content covers how to cite sources, types of plagiarism with their repercussions, and the variations between plagiarism and paraphrasing.

**Table 2:** Existing library games around the world.

Game name	Description of library game	University/college library	Educational content
Nightmare on Vine Street	In this game, the player is secured inside the library around evening time and must satisfy zombies with assets found in the library to get away [14,64].	University of Tennessee at Chattanooga, USA	The game improves the information literacy of students.
Within Range	In this game, the player has to place books according to the call number before the allocated time is up [14,64,65].	Carnegie Mellon University, USA	Library staff learns how to store books.
Defense of Hidgeon	At the beginning of this game, students are instructed to start library research while navigating, looking, and finding assets. During the game, students have to go to distinctive libraries and search web for answers [64,66].	University of Michigan, USA	Students learn how to conduct research using library resources.
Secret Agents in the Library	The player assumes the role of a secret agent in this online game. The objective of the game is to protect the library from an invader by answering questions as well as fetching assets from within the library [14].	Lycoming College, USA	The game improves the information literacy of students, and students learn how to access the library website.
It’s Alive	Here, the player acts as a crazy scientist endeavoring to obtain body parts to build an animal, and to obtain these body parts, the player has to correctly respond to a sequence of questions [14,65].	Lycoming College, USA	Players are able to find out about biology research methods.
Get a Clue	Students have to solve a crime by visiting assigned areas and getting rid of suspects and library resources in the game [64].	Utah Valley University, USA	Students are able to navigate around the university library.
LibraryCraft	Students can visit various pages of the library website. Students need to find a book in their catalogue and use the author’s name as a password to show that they have completed the task [14,67].	Utah Valley University, USA	Students find out about the library resource/assets and how to manage research.

#### Gaps

Although there have been attempts by different game developers and researchers to create educational games for teaching students what plagiarism is, the following issues are yet to be addressed:

- Story and gaming environment: For this, we proposed a 2-sided storyline that allows a student to play as a good guy or a bad guy, detecting plagiarism or plagiarizing and escaping being caught, respectively, within the game.
- Replayability: To the best of our knowledge, none of the existing games has the ability to present the player with new problems every time they play. We have written new algorithms for this task.
- First local solution: As far as we can tell, no South African university has an educational game for teaching plagiarism.

These issues are addressed in the design of Plagi-Warfare, discussed in the Methods section, covering the design aspects of Plagi-Warfare, such as the game flow, story development, and other design considerations.

### Future Work

We are currently busy digitizing several topics offered by the university’s library to make them more attractive to students. Educational games are at the center of this digitization; hence, we anticipate more work in this space in the future. In the future, we will also open up the evaluation of Plagi-Warfare to the entire student population of our university. Given that we have over 40,000 students, we hope to gain more insights into what we can improve about this game.

### Conclusion

In this paper, we presented a new educational game that teaches students about plagiarism by using a new crime story and an immersive 3D gaming environment representing the university libraries of the University of Johannesburg. To do this, we allowed players to play as either a mafia member or a detective and supplied them with procedurally generated quizzes that ensure that the game remains replayable. We demonstrated the first version of the game and presented the evaluation of the game. The responses from the students show that the game can be used to learn about plagiarism and that they would recommend it to others. Plagi-Warfare addresses a major gap in South Africa, as there is no such game in any South African university, and worldwide with its unique storyline and its environment that allow the global community to experience our libraries at the University of Johannesburg. Given the increasing appeal of video games among digital natives, the use of an immersive game about plagiarism is expected to give more students the motivation and eagerness to learn about plagiarism.

## Abbreviations

APB: Auckland Park Bunting Road
APK: Auckland Park Kingsway
DFC: Doornfontein
E-R: entity-relationship
FoV: field of view
IDE: Integrated Development Environment
NPC: nonplayer character
